# Supplementary material for: Causal Relationship between Aging and Anorexia Nervosa: A White-Matter-Microstructure-Mediated Mendelian Randomization Analysis
Source: Biomedicines. 2024 Aug 16;12(8):1874. doi: 10.3390/biomedicines12081874 (PMC11351342; doi:10.3390/biomedicines12081874)
Supplement: Supplementary file 1 [file biomedicines-12-01874-s001.zip › Supplementary Figure/Figure S8/Leaveoneout plot of MR analysis for TL to ubm-b-2035.pdf]

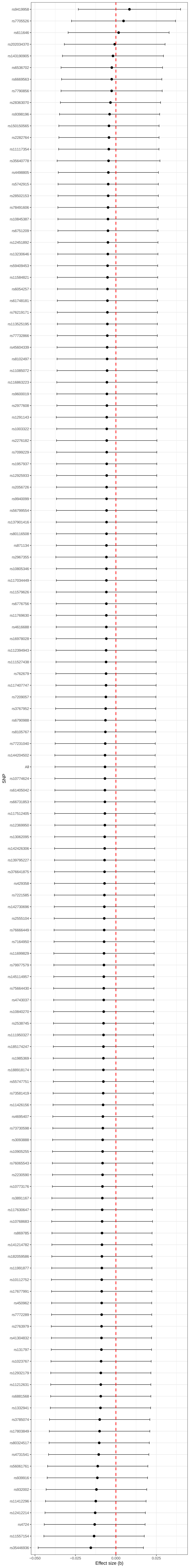

SNP
Effect size (b)
-0.050
-0.025
0.000
0.025
rs9419958
rs7705526
rs611646
rs202034370
rs143190905
rs6536702
rs6669563
rs7790856
rs28363070
rs9398196
rs150150565
rs2282764
rs11117354
rs35640778
rs4498805
rs5742915
rs28502153
rs78491606
rs10645387
rs6751209
rs12451892
rs13230646
rs59409453
rs11584821
rs6054257
rs61748181
rs76219171
rs113525195
rs77732866
rs45604339
rs8102497
rs11085072
rs116863223
rs9600019
rs2977608
rs1291143
rs1003322
rs2276182
rs7099229
rs1957937
rs12925933
rs2056726
rs9940099
rs56799554
rs137901416
rs80116508
rs871134
rs2967355
rs10805346
rs117034449
rs11579626
rs6776756
rs11769630
rs4616688
rs16978028
rs112394943
rs111527438
rs762679
rs117407747
rs7209057
rs3767952
rs6790988
rs8105767
rs77231040
rs144204502
All
rs10774624
rs61405042
rs66731853
rs117512405
rs12369950
rs13062095
rs142426306
rs139795227
rs376641875
rs429358
rs7221585
rs142730696
rs2555104
rs76666449
rs7164950
rs11699829
rs79977579
rs145114957
rs75664430
rs4743037
rs10840270
rs2538745
rs111950327
rs185174247
rs1985369
rs188918174
rs55747751
rs73581419
rs11426156
rs4695407
rs73730598
rs3093888
rs10905255
rs76065543
rs2230590
rs10773176
rs3891167
rs117630647
rs10768683
rs869785
rs141214782
rs182059586
rs11991877
rs10112752
rs17677991
rs450962
rs7772289
rs2763979
rs41304832
rs131797
rs1023767
rs12932179
rs11212631
rs6881568
rs1332941
rs3785074
rs17803849
rs80324517
rs4731541
rs56061761
rs939916
rs932002
rs11412296
rs12412214
rs4724
rs11557154
rs35446936
